# A guidance of model selection for genomic prediction based on linear mixed models for complex traits

**DOI:** 10.3389/fgene.2022.1017380

**Published:** 2022-10-05

**Authors:** Jiefang Duan, Jiayu Zhang, Long Liu, Yalu Wen

**Affiliations:** ^1^ Department of Health Statistics, School of Public Health, Shanxi Medical University, Taiyuan, Shanxi, China; ^2^ Department of Statistics, University of Auckland, Auckland, New Zealand

**Keywords:** alzheimer’s disease, brain structure, genetic architecture, linear mixed model, model selection, risk prediction

## Abstract

Brain imaging outcomes are important for Alzheimer’s disease (AD) detection, and their prediction based on both genetic and demographic risk factors can facilitate the ongoing prevention and treatment of AD. Existing studies have identified numerous significantly AD-associated SNPs. However, how to make the best use of them for prediction analyses remains unknown. In this research, we first explored the relationship between genetic architecture and prediction accuracy of linear mixed models *via* visualizing the Manhattan plots generated based on the data obtained from the Wellcome Trust Case Control Consortium, and then constructed prediction models for eleven AD-related brain imaging outcomes using data from United Kingdom Biobank and Alzheimer’s Disease Neuroimaging Initiative studies. We found that the simple Manhattan plots can be informative for the selection of prediction models. For traits that do not exhibit any significant signals from the Manhattan plots, the simple genomic best linear unbiased prediction (gBLUP) model is recommended due to its robust and accurate prediction performance as well as its computational efficiency. For diseases and traits that show spiked signals on the Manhattan plots, the latent Dirichlet process regression is preferred, as it can flexibly accommodate both the oligogenic and omnigenic models. For the prediction of AD-related traits, the Manhattan plots suggest their polygenic nature, and gBLUP has achieved robust performance for all these traits. We found that for these AD-related traits, genetic factors themselves only explain a very small proportion of the heritability, and the well-known AD risk factors can substantially improve the prediction model.

## Introduction

Recent studies have shown that Alzheimer’s disease (AD) has become the fifth cause of death for Americans aged 65 and above, and it is predicted that the number of AD patients in the United States will increase to 13.8 million by 2060 (Association A.S., 2022) and one in 85 people worldwide will have the disease by 2050 (Cacace et al., 2016). Age is one of the most important risk factors for AD. With the growth of the elderly population, the family and social burden caused by the care and nursing of AD patients will become increasingly heavy in the future. AD, the most common cause of dementia, is an irreversible neurodegenerative disorder characterized by progressive cognitive and memory impairment that is enough to interfere with daily life (Guerreiro et al., 2012; Cacace et al., 2016; Zhu et al., 2019). At present, only symptomatic patients can be treated, which cannot prevent the further deterioration and development of AD (Jiang et al., 2012). However, the preclinical stage of AD is as long as 7 years (Bischkopf et al., 2002), and detecting high risk population during this pre-clinical stage could postpone the progression to AD.

Brain imaging genetics can discover neural mechanisms associated with AD by combining genetic information and neuroimaging data of the same subjects. It is anticipated that the investigation of brain-related imaging traits can substantially facilitate the understanding of the pathogenesis of AD and provide guidance for its treatment and prevention. Currently, many different types of imaging, such as Magnetic Resonance Imaging (MRI), Positron Emission Tomography (PET), and Diffusion Tensor Imaging (DTI), are used to facilitate AD diagnosis. They contain both confirmatory and complementary information, showing the changes of brain structure of patients from different perspectives. For example, DTI provides the local microscopic characteristics of water diffusion; structural MRI can be used to describe brain atrophy; functional MRI characterizes hemodynamic responses related to neural activity; and PET measures metabolic patterns in the brain (Wee et al., 2012). Studies have shown that some brain regions, notably the *Hippocampus*, Parahippocampal gyrus, Cingulate, and Entorhinal cortex, are reduced in patients with mild cognitive impairment (MCI) and AD. Researchers have found that both gray matter and white matter have significantly decreased for individuals who underwent transition from MCI to AD (Misra et al., 2009). Atrophy of brain volume in these regions can reflect the stage of disease development and predict the progression of AD (Basaia et al., 2019). It has become a focus to identify patients with brain diseases based on brain imaging. At present, studies have shown that age, gender and education level are the most important predictors for AD (Rogaeva, 2002; Dumitrescu et al., 2019). Over the past decades, genomics data, such as single nucleotide polymorphisms (SNPs), have become increasingly available, and they have provided valuable data resources for investigating and predicting AD from genetic perspective (Clark and van der Werf, 2013). It has been reported that human brain structure is highly heritable and genetic factors account for 58–74% of the risk of AD (Carmelli D et al., 1998; Braskie et al., 2011; Satizabal et al., 2019). Therefore, incorporating genetic factors into the investigation of AD and AD-related traits hold great promise in better understanding of AD.

Linear mixed models (LMMs) and their extensions have become the method of choice for the risk prediction analysis using genomic data. Their fundamental assumption is that genetically similar individuals have similar phenotypes, and the genetic similarities are usually measured by SNPs. The differences in existing LMM-based risk prediction models primarily lie in the assumption of the underlying disease model that can be broadly categorized into two categories, including the sparse and polygenic models. The sparsity regression models are commonly used for prediction when it is believed that the phenotypes are caused mainly by a limited number of SNPs with moderate to large effects and the rest SNPs are noise. Bayesian mixture models that utilize different prior to reflect the assumed sparsity are one of the commonly used methods. For example, BayesR (Moser et al., 2015) and Bayesian Sparse Linear Mixed Model (BSLMM) (Visscher et al., 2013) set the effect sizes of some SNPs to be zero to introduce sparsity. While sparsity regression models can be helpful for diseases that are caused mainly by SNPs with moderate to large effects (e.g., Type I diabetes), for most complex traits, the known disease-associated markers only explain a small proportion of heritability (Marigorta et al., 2018) and the sparse models are obviously not applicable. As reported, the heritability of human height estimated by common SNPs is larger than that obtained from significant SNPs (Yang et al., 2010). The polygenic models that assume a large number of SNPs have small to moderate predictive effects have been used extensively for complex trait prediction. The genomic best linear unbiased prediction (gBLUP) (Clark and van der Werf, 2013), the seminal work in polygenic risk model, has been adopted widely in genomic risk prediction studies (Alvarenga et al., 2020; Bermann et al., 2021; Zhang et al., 2021). The gBLUP assumes that effect sizes from all SNPs follow the same normal distribution, and predicts the phenotypes of interest based on the information provided by all SNPs. It was then extended to accommodate more complex disease model assumptions. For example, MultiBLUP (Speed and Balding, 2014) allows SNPs from different genomic regions having different effect size distributions, and the latent Dirichlet process regression (DPR) (Zeng and Zhou, 2017) uses a Dirichlet process to allow the effect sizes have any form of distributions instead of assuming the normality. While polygenic models have achieved various levels of success and have been used widely for complex traits prediction in practice, their performance still highly depends on the underlying disease model.

It is well accepted that there are no universal best models for risk prediction analysis based on genomic data, and the underlying genetic architecture is the key factor that determines the predictive performance of these LMM-based models. Existing studies that have identified numerous AD-associated SNPs and genes (Daw et al., 2000; Sims and Williams, 2016). However, how to choose the most appropriate statistical models for the prediction of AD and its related traits is unknown. It is still not clear the exact amount of differences among these commonly used risk prediction models given different underlying genetic mechanisms. Therefore, in this research, we first explored the relationship between disease model and prediction performance for polygenic models (i.e., gBLUP, MultiBLUP and DPR) and sparsity model (i.e., BayesR) using the Wellcome Trust Case Control Consortium (WTCCC) dataset that has seven diseases with a broad spectrum of genetic architecture. We then build risk prediction for a range of AD-related brain imaging traits using the United Kingdom Biobank data (UKB), and further validated these models using the data obtained from the Alzheimer’s disease neuroimaging initiative (ADNI). Through the above comprehensive analyses, we have provided guidance on how to select an appropriate prediction model for a given dataset. We also explored the genetic architecture as well as appropriate methods and modeling strategies for AD-related brain imaging traits, so as to identify the high-risk population of AD at an early stage.

## Methods and materials

In the following sections, we first described the datasets that we analyzed, including WTCCC, UKB and ADNI, and their corresponding quality control. We then briefly overviewed the technical details of the four prediction models (i.e., gBLUP, MultiBLUP, DPR and BayesR), and finally we detailed the risk prediction analyses for eleven AD-related brain imaging traits in our study.

### Data description and quality control

WTCCC contains seven complex diseases with each having a sample size of about 2000. These seven diseases include bipolar disease (BD), coronary artery disease (CAD), Crohn’s disease (CD), rheumatoid arthritis (RA), type 1 diabetes (T1D), type 2 diabetes (T2D), and hypertension (HT) (Feng and Zhu, 2010). WTCCC also has a shared control group that consists of 1958 Birth Cohort (*n* = 1,500) and United Kingdom Blood Service sample (*n* = 1,500). Since the seven diseases collected by WTCCC covers a broad spectrum of genetic architecture, we used this dataset to investigate the relationship between genetic architecture and the accuracy of risk prediction models. DNA samples were drawn from study participants and they were analyzed using the Affymetrix 500 K platform (Gershon et al., 2008). Genotypes were called by the CHIAMO algorithm and directly downloaded the WTCCC website (https://www.wtccc.org.uk/). Only autosome SNPs were considered in our analysis. For its quality control, SNPs were excluded if they met any of the following conditions: 1) missing rate >2%, 2) minor allele frequency (MAF) < 1%, 3) *p*-value for Hardy Weinberg equilibrium (HWE) test less than 1e-10 and 1e-6 for cases and controls, respectively (Marees et al., 2018). Individuals with missing rate larger than 2% were also removed. Each case dataset was combined with the controls, and common SNPs between cases and controls were retained. For each combined data, we applied further quality control to remove SNPs with missing rate >2%, MAF <5%, *p* < 1e-6 from HWE as well as *r* ^2^ > 0.2 from linkage disequilibrium and subjects with missing rate >2%. After the quality control, the number of individuals for each disease ranged from 4,862 to 4,926, and the number of SNPs ranged from 67,281 to 68,412 (Supplementary Table S1).

Genetic and brain imaging data collected from both UKB (Bycroft et al., 2018) and ADNI (Wyman et al., 2013) are used for risk prediction studies. UKB is the largest prospective cohort study to date, collecting health-related information including demographic, lifestyle indicators, biomarkers in blood and urine, brain imaging, and genetic information from nearly 500,000 subjects aged from 40 to 69 in the United Kingdom. They have collected brain imaging data covering structural, diffusion and functional imaging, which provide detailed information for the brain structure. In addition, known risk factors for AD, such as age, gender, and education (coded according to 21,003-0.0, 6,138–0.0 and 31-0.0) have also been collected. Blood DNA samples were obtained from study participants. 49,950 samples were analyzed using the Affymetrix Applied Biosystems United Kingdom BiLEVE Axiom Array and the remaining 438,427 samples were processed with the Applied Biosystems United Kingdom Biobank Axiom Array. About 95% of the markers are the same for these two arrays (825,927 markers), and we used these markers for our analyses.

ADNI (Wyman et al., 2013), including ADNI1, ADNI2, ADNIGO and ADNI3, is a large-scale longitudinal study designed to find the AD-related biomarkers and improve the clinical diagnosis of AD. At baseline, demographic variables (e.g., age, sex and education), brain imaging outcomes including MRI (e.g., structural, diffusion weighted imaging, perfusion and resting state sequences) and PET, biomarkers, and genetic information from each participant were collected. DNA samples were obtained and analyzed using Illumina’s non-CLIA whole genome sequencing. In our analysis, we focused on the baseline brain imaging and genotype data collected from all ADNI study participants except ADNI3.

Similar to WTCCC data, we only focused on autosome SNPs for both ADNI and UKB. Since population structure can be a serious confounder, only white and non-Hispanic individuals were retained through principal component analysis (Marchini et al., 2004). This can ensure that ADNI and UKB have the same population structure, which allows for external validation. For both ADNI and UKB, we first removed SNPs when 1) call rate <90%; 2) *p* < 1e-6 from HWE; or 3) MAF <5%. We also excluded individuals with call rate <90%. For the remaining samples, missing values for SNPs were imputed using the default procedures in plink 1.9. With imputed data, we further filtered out SNPs with call rate <99%, HWE < 1e-6 and MAF <5%, as well as excluded individuals with call rate <99%. After the quality control, 738 subjects with 6,250,600 SNPs were remained in ADNI. 488,371 subjects with 211,127 SNPs were remained in UKB. Finally, we extracted a total of 202,840 common SNPs from ANDI and UKB for subsequent modeling.

We focused on brain imaging traits from both UKB and ANDI studies. These traits include subcortical volumes (hippocampus, accumbens, amygdala, caudate, *pallidum*, putamen, thalamus), the volumes of gray matter, white matter and brainstem+4^th^ ventricle from T1 structural brain MRI, and the volume of white matter hyperintensities from T2-weighted brain MRI. The sample sizes for each phenotype in both UKB and ADNI studies are summarized in Supplementary Table S2.

### The technical details of risk prediction models

gBLUP is one of the most widely used genomic risk prediction model. It assumes that the effect sizes for all SNPs follow a normal distribution, and models the outcomes as $y_{i}=X_{i}\beta +\underset{j}{\sum }g_{ij}\gamma_{j}+\epsilon_{i}$ , where $X_{i}$ is the demographic variables (e.g., age and gender), $g_{ij}$ is the genotype for the *j*th marker for individual i, $\gamma_{j}∼N\left(0,\sigma_{g}^{2}\right)$ is predictive effect from the *j*th marker, and $\epsilon_{i}∼N\left(0,\sigma_{0}^{2}\right)$ . It is straightforward to see that $Y∼N(X\beta ,\;K\sigma_{g}^{2}+I\sigma_{0}^{2}$ ), where $K=GG^{T}$ is the genomic similarity matrix (GSM) estimated based on all SNPs. Various software has implemented the gBLUP method, and we used the LDAK software with its default settings for our analysis.

MultiBLUP can be viewed as an extension of gBLUP, where SNPs from different genomic regions are allowed to have different effect size distributions. It splits the genome into *R* regions and models the outcomes as $y_{i}=X_{i}\beta +\overset{R}{\underset{r}{\sum }}\underset{j\in S_{r}}{\sum }g_{irj}\gamma_{rj}+\epsilon_{i}$ , where $S_{r}$ is the set of all genetic markers in region *r*, $g_{irj}$ is the *j*th genotype for the *i*th individual in region r, and $\gamma_{rj}∼N\left(0,\sigma_{r}^{2}\right)$ with $\sigma_{r}^{2}$ could be different among regions. Consequently, $Y∼N(X\beta ,\;\overset{R}{\underset{r}{\sum }}K_{r}\sigma_{r}^{2}+I\sigma_{0}^{2}$ ), where $K_{r}=G_{r}G_{r}^{T}$ and $G_{r}$ are a matrix of all SNPs within region *r*. gBLUP is a special case of MultiBLUP, where all $\sigma_{r}^{2}$ are the same. MultiBLUP defines regions either empirically or based on some annotations. Therefore, some of the regions can have similarly $\sigma_{r}^{2}$ and could be combined. The adaptive MultiBLUP (AMB) that combines regions with similar $\sigma_{r}^{2}$ can reduce the computational complexity for the LMM model, and thus is used more often in practice. Therefore, for our analysis, we adopted the AMB, which is implemented in the LDAK software. We used its default setting, where the genome was divided into chunks with the size of 75,000 base pairs. After the LR-ratio test, regions were formed by merging each chunk with *p* < 10^–5^ with its neighboring regions that have *p*-value less than 10^–2^.

DPR models the phenotypes using the same model as gBLUP, except the variance of effect sizes is modelled using a non-parametric Dirichlet process $\sigma^{2}∼G,\;G∼DP\left(H,\lambda \right)$ , where *H* is the base distribution and λ is the concentration parameter that describes how the distribution *G* deviates from the base distribution *H*. DPR uses the inverse Gamma as the base distribution and uses data at hand to infer λ . With the stick-breaking constructive representation and using the same concentration parameter, the effect size $\gamma_{j}$ is effectively modelled as:

$$\gamma_{j}∼\overset{+\infty }{\underset{k=1}{\sum }}\pi_{k}N\left(0,\sigma_{k}^{2}\right)\;and\;\pi_{k}=v_{k}\overset{k-1}{\underset{l=1}{\prod }}\left(1-v_{l}\right),v_{k}∼Beta\left(1,\lambda \right).$$ (1)

With the infinite normal mixture prior, DPR can approximate a large class of unimodal distribution, and thus can robustly predict traits with various genetic architecture. Its parameters can be estimated by the traditional Markov Monte Carlo Chain (MCMC) algorithm, denoted as DPR. MCMC, or by the mean variational Bayesian (VB) approximation algorithm, denoted as DPR. VB. Both DPR. MCMC and DPR. VB are implemented in the DPR software. As demonstrated in Zeng et al. (Zeng and Zhou, 2017), DPR. VB can achieve similarly level of prediction accuracy with much faster computational speed than DPR. MCMC. Therefore, for all our analyses, we used DPR.VB. In addition, the default implementation of DPR does not take covariates into account. To consider their contribution, we adopted a commonly used iterative two-step procedure (Bolormaa et al., 2017), where the traits were first regressed on covariates and then the residuals were analyzed using the DPR model. These two steps continued until convergence.

BayesR (Moser et al., 2015) predicts the phenotypes using the same model as gBLUP, except that the effect size $\gamma_{j}$ is assumed to follow a normal mixture:

$$\gamma_{j}∼\pi_{1}N\left(0,{0\sigma }_{g}^{2}\right)+\pi_{2}N\left(0,{10^{-4}\sigma }_{g}^{2}\right)+\pi_{3}N\left(0,10^{-3}\sigma_{g}^{2}\right)+\pi_{4}N\left(0,10^{-2}\sigma_{g}^{2}\right)$$ (2)

where $\overset{4}{\underset{i}{\sum }}\pi_{i}=1$ . Apparently, the mixture proportions determine the sparsity of the model (Erbe et al., 2012). Gibbs sampling is used for inference in BayesR (Bolormaa et al., 2017). The posterior inclusion probability can be used to infer how likely a SNP is predictive. BayesR can be viewed as a sparsity regression model and it can have better prediction performance than polygenic models when phenotypes are affected by several SNPs with large effects. We used BayesR implemented in the BayesR software in our analysis. Rather than using their default setting that can be extremely computationally demanding, we set the Markov chain length to be 4,000, with the first 800 samples discarded as burn-in and posterior estimates of parameters are based on 3,000 samples drawing every 10th sample after burn in. These parameters were chosen from a set of Markov chain lengths that provided the highest Pearson correlation.

All the above four models can be viewed as a linear mixed model, and differ mainly in the assumption on effect size distribution (Table 1). We chose to analyze our traits using these methods primarily because of their popularity and their capacity in modeling traits with various genetic architecture (e.g., the sparsity model and the infinitesimal model). We believe these models can provide us an insight into how to best model AD-related traits given their unknown underlying genetic mechanisms.

**TABLE 1:** The prior distributions of random effects under different methods.

Methods	Effect size distribution
gBLUP	$\gamma_{j}∼N\left(0,\sigma_{g}^{2}\right),\forall j$
MultiBLUP	$\gamma_{rj}∼N\left(0,\sigma_{r}^{2}\right),\;r\in \left\{1,2,\cdots ,\;R\right\}$
DPR	$\gamma_{j}∼\overset{+\infty }{\underset{k=1}{\sum }}\pi_{k}N\left(0,\sigma_{k}^{2}\right){,\;\pi }_{k}=v_{k}\overset{k-1}{\underset{l=1}{\prod }}\left(1-v_{l}\right),{\;v}_{k}∼Beta\left(1,\lambda \right)$
BayesR	$\gamma_{j}∼\pi_{1}N\left(0,{0\sigma }_{g}^{2}\right)+\pi_{2}N\left(0,{10^{-4}\sigma }_{g}^{2}\right)+\pi_{3}N\left(0,10^{-3}\sigma_{g}^{2}\right)+\pi_{4}N\left(0,10^{-2}\sigma_{g}^{2}\right)\;with\;\overset{4}{\underset{i}{\sum }}\pi_{i}=1$

### The analysis workflow

For the analysis of WTCCC, we first conducted genome-wide association studies to explore the genetic architecture of each disease, and then used the four methods (i.e., gBLUP, AMB, DPR, and BayesR) to build prediction models for them. Specifically, GWAS was conducted using the plink software (version 1.9), where the default setting (i.e., logistic regression with SNPs assumed to have additive effects) is used. We visualized the results using the ggplot2 package in R (version 4.2.0). We used simple linear regression for quantitative traits (i.e., eleven brain imaging traits in UKB) and logistic regression for binary traits (i.e., seven diseases in WTCCC) under the additive effect assumption. To avoid overfitting and chance finding, we randomly selected 90% of samples from each data to build prediction models and used the remaining for validation. We repeated this process 20 times and reported the average Area Under Curve (AUC) calculated based on the validation samples.

For the analysis of AD-related brain imaging traits, we first conducted the GWAS, where plink 1.9 software with the default settings (i.e., simple linear regression model with SNPs assumed to have additive effects) was used. We used the UKB data to build the models and the ADNI data for external validation. For UKB data, we first estimated the heritability for each trait using the Genome-wide complex trait analysis (GCTA) software, and then conducted an association test to explore their genetic architecture. For prediction modeling, we considered prediction models with and without covariates. Specifically, for the models without covariates, we used UKB data to build the prediction models and reported the prediction accuracy that includes Pearson correlation and mean square error (MSE) based on 20-fold cross-validation, and further validated the model using ADNI data. For the model with covariates, we included well-known AD-related demographic risk factors (i.e., age, gender and education). We used the same procedures as the model without covariates and reported both the cross-validation and external validation accuracies.

## Results

### The exploration of disease model and accuracies of risk prediction models

**FIGURE 1:**
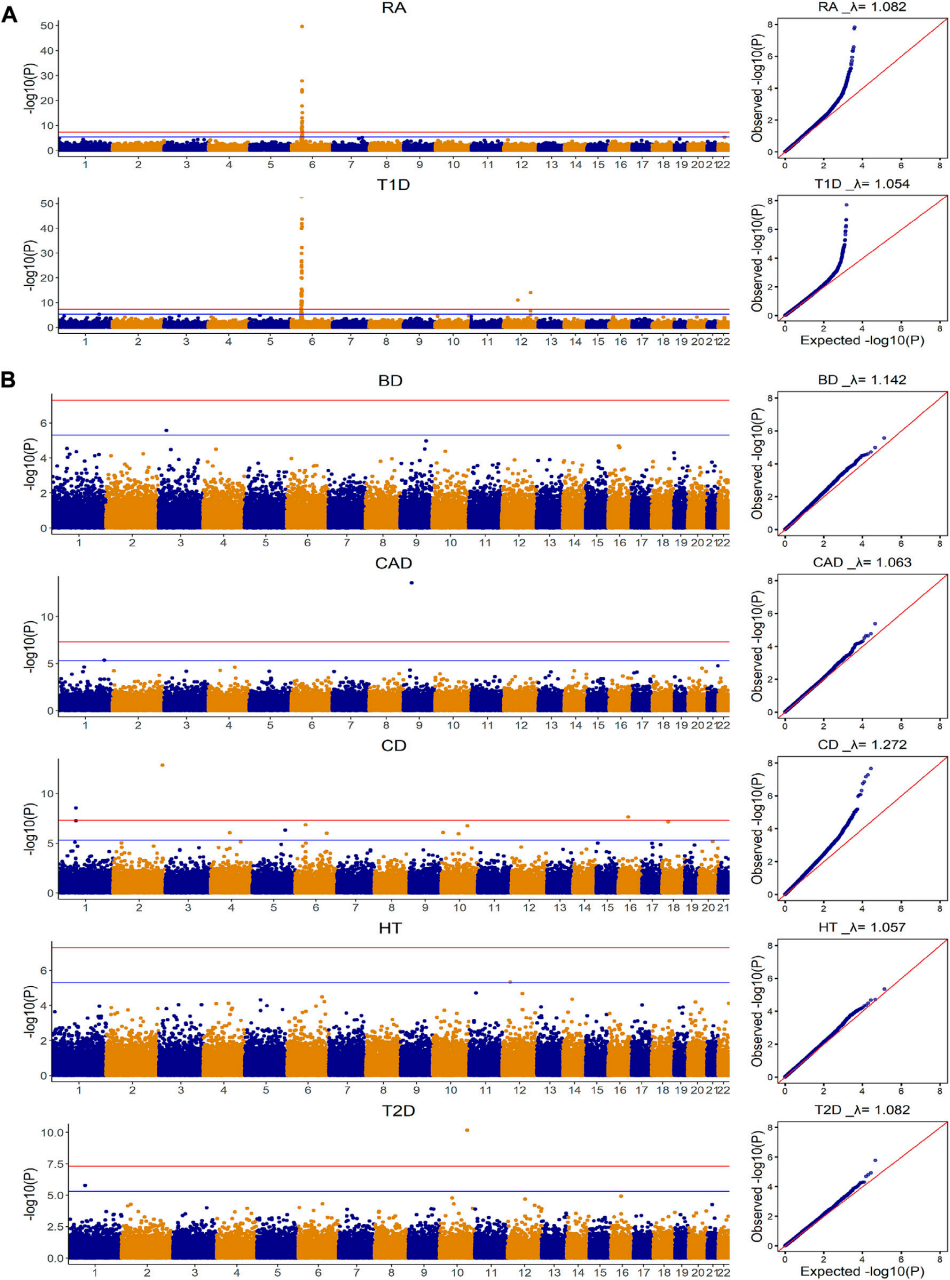
Manhattan and Quantile-Quantile plots for seven diseases in WTCCC based on additive model for Genome-wide association analysis. CD: Crohn’s disease; RA: Rheumatoid arthritis; T1D: Type 1 diabetes; BD: Bipolar disease; CAD: Coronary artery disease; HT: Hypertension; T2D: Type 2 diabetes. The λ closer to 1 indicates appropriate control of population structure **(A)** This group included T1D and RA, where Manhattan plots suggested dense and spiked signals at the genome-wide significance level (i.e., *p* < 5 × 10^–8^) **(B)** This group included BD, CAD, CD, HT and T2D, where their Manhattan plots indicated only few SNPs achieved significance at a suggestive genome-wide significance level (i.e., *p* < 5 × 10^–6^).

Figure 1 summarizes the genome-wide association results for the seven diseases in the WTCCC data. Based on the Manhattan plots, we divided the seven diseases into two groups. The first group included T1D and RA, where Manhattan plots suggested dense, clustered and spiked signals for each disease (i.e., *p* < 5 × 10^–8^). The Manhattan plots indicated that these diseases have SNPs with larger than commonly assumed small-to-moderate effects, and thus models that allow SNPs from different genetic regions having different effect sizes have the potential to outperform the models that only assume infinitesimal effects. The second group included BD, CAD, CD, HT and T2D, where their Manhattan plots showed only a few SNPs achieved significance at a suggestive association threshold (i.e., *p* < 5 × 10^–6^). From the Manhattan plots, it is quite unlikely that the sparsity regression model alone can capture all the predictive effects for these diseases. While the genetic etiology is unknown for most of common diseases, making it hard to choose appropriate prediction models, the simple Manhattan plots can provide valuable insights on which model assumptions are more appropriate for the genomic risk score calculation.

**FIGURE 2:**
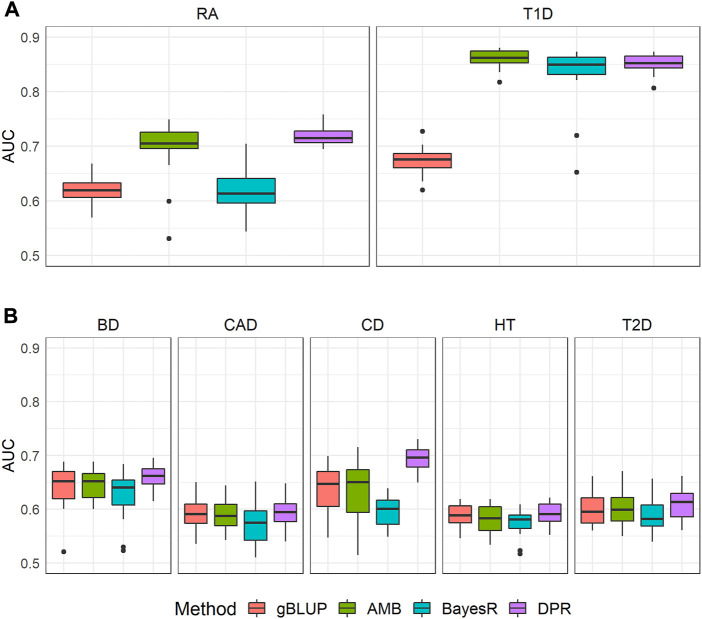
The area under the curve (AUC) for seven diseases in WTCCC. CD: Crohn’s disease; RA: Rheumatoid arthritis; T1D: Type 1 diabetes; BD: Bipolar disease; CAD: Coronary artery disease; HT: Hypertension; T2D: Type 2 diabetes. Methods include genomic best linear unbiased prediction (gBLUP), adaptive MultiBLUP (AMB), the latent Dirichlet process regression (DPR) and BayesR **(A)** This group included T1D and RA, where Manhattan plots suggested dense and spiked signals at the genome-wide significance level (i.e., *p* < 5 × 10^–8^) **(B)** This group included BD, CAD, CD, HT and T2D, where their Manhattan plots indicated only few SNPs achieved significance at a suggestive genome-wide significance level (i.e., *p* < 5 × 10^–6^).

The prediction performance for these seven diseases based on four methods is shown in Figure 2. Consistent with our exploration, for both T1D and RA where the Manhattan plots showed significant SNPs, the gBLUP model assuming all SNPs have the same effect size distribution performed the worst, whereas AMB and DPR achieved the highest AUC. BayesR had similar AUC as that of AMB and DPR for T1D, but it performed much worse than AMB and DPR for the prediction of RA. This suggested that although significant SNPs contributed substantially to the prediction of both RA and T1D, some SNPs with small to moderate predictive effects also contribute to risk of RA. Since BayesR sets the small effect size to 0, it loses the capacity in capturing their contributions. On contrary, both DPR and AMB do not force the effect size to be zero, and thus are likely to capture their contributions.

For the other five diseases (BD, CAD, CD, HT and T2D), the model assuming only the sparsity effects (i.e., BayesR) performed the worst. For the comparison of the remaining methods (i.e., gBLUP, AMB and DPR), we noticed that they performed very similarly for BD, CAD, HT and T2D. For CD, the DPR appeared to be better than both gBLUP and AMB. As shown in Manhattan plots (Figure 1), for BD, CAD, HT and T2D, it is likely the infinitesimal effect model assumption is appropriate, and thus models that are designed to capture them have better performance as compared to the sparsity regression model. For CD that had a few isolated SNPs achieved the suggestive genome-wide significant level, the model (i.e., DPR) that can capture both the infinitesimal effects and spiked isolate predictive effects tended to higher AUC. gBLUP only assumes the infinitesimal effect model, and thus is not capture of capturing the predictive effects from these isolated markers for CD. Similarly, while AMB can capture predictive effects from SNPs with different distributions, it requires these SNPs located nearby. Since the Manhattan plot for CD in Figure 1 clearly showed that these SNPs are located far away, AMB cannot effectively model them.

The computational resources needed for each of the four prediction models were summarized in Supplementary Table S3. The average computational time for gBLUP, AMB, DPR and BayesR was 0.32, 0.89, 18.24 and 5.60 h, respectively. This was 2.81, 57.23 and 17.57 times of the computational time required by gBLUP. The memory consumption for gBLUP, AMB, DPR and BayesR was 0.38.0.33, 4.89 and 0.32 GB on average, respectively. This was 0.86, 12.87 and 0.84 times of that required by gBLUP. Apparently, gBLUP was the most computationally efficient model, whereas DPR required the most resources. The exploration of the relationship between genetic architecture (approximated by the Manhattan plot) and accuracy of the commonly used prediction models suggests that the models (i.e., DPR and AMB) that can model both sparse effects as well as infinitesimal effects perform better for diseases with highly significant SNPs. The gBLUP model would not be recommended under such conditions. However, for diseases that have no apparent associated markers, the simplest gBLUP model can achieve similar levels of performance with much more computational efficiency.

### The prediction of brain imaging traits

**FIGURE 3:**
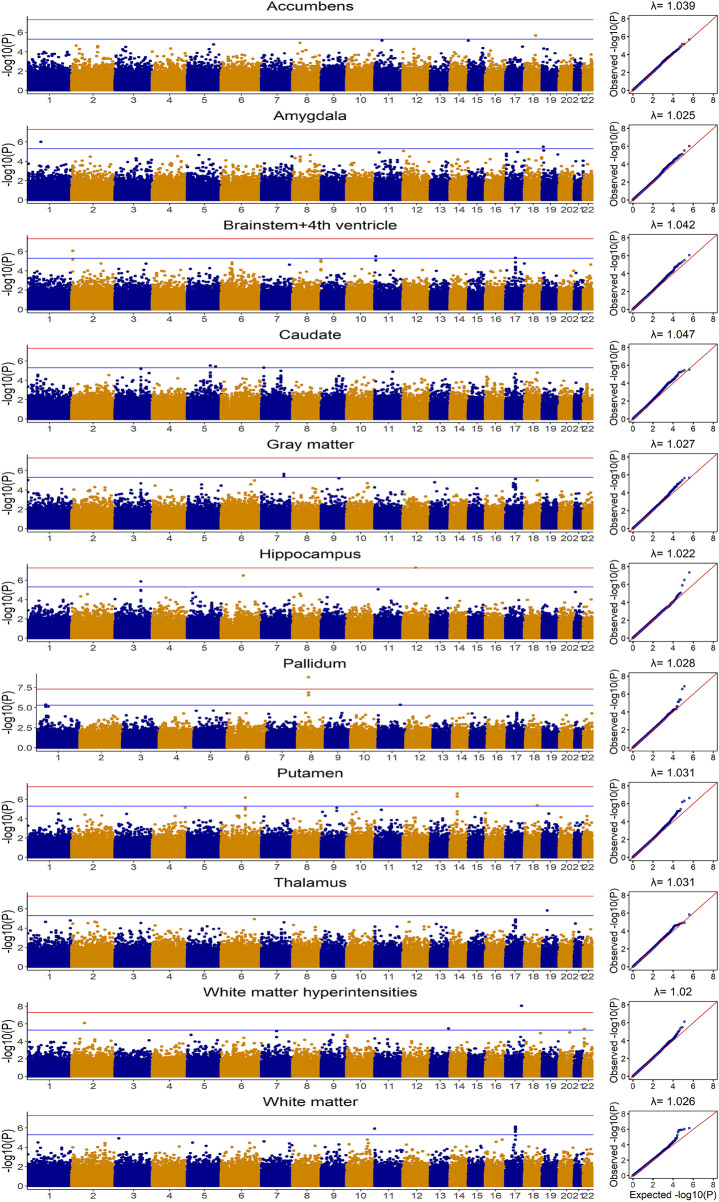
Manhattan and Quantile-Quantile plots for eleven brain imaging traits in UKB based on additive model for Genome-wide association analysis.

The distributions for brain imaging traits in UKB and ADNI as well as their estimated heritability based on UKB were shown in Table 2 and the demographic information was summarized in Supplementary Table S4. The Manhattan and Quantile-Quantile plots for these eleven brain imaging traits for UKB were shown in Figure 3. The trends in Manhattan plots for all AD-related brain imaging traits were similar to BD, CAD, HT and T2D in the WTCCC data, suggesting the infinitesimal effect model assumption was more appropriate for their analysis. As a result, we expected the simplest gBLUP model was sufficient in their modeling.

**TABLE 2:** The distributions of eleven brain imaging traits in UKB and ADNI as well as their heritability based on UKB.

Phenotype	Mean ± SD (mm^3^)		Heritability^b^
	UKB	ADNI	
Hippocampus	7,722.46 ± 867.13	7,001.53 ± 1,091.68	0.1412
Accumbens	909.58 ± 207.32	951.48 ± 172.96	0.2618
Amygdala	2,535.28 ± 435.41	2,694.41 ± 468.08	0.1486
Caudate	6,964.53 ± 844.13	6,914.15 ± 1,030.63	0.3936
Pallidum	3,571.99 ± 452.18	3,025.72 ± 395.92	0.1516
Putamen	9,678 ± 1,143.54	9,399.75 ± 1,183.19	0.2324
Thalamus	15,395.64 ± 1,455.59	12,351.39 ± 1,357.41	0.2036
Gray matter	617,733.3 ± 55,268.39	584,396 ± 55,343.19	0.2059
White matter	553,167 ± 62,263.83	476,159.4 ± 60,155.71	0.1967
White matter hyperintensity^a^	4,187.09 ± 5,679.05	6,838.04 ± 10,275.04	0.1343
Brainstem+4th ventricle	22,933.21 ± 2,705.64	20,797.34 ± 2,323.53	0.2823

a White matter hyperintensity, logarithm to base 10 was performed to make it to be approximately normal distribution.

b The heritability was estimated based on UKB.

**FIGURE 4:**
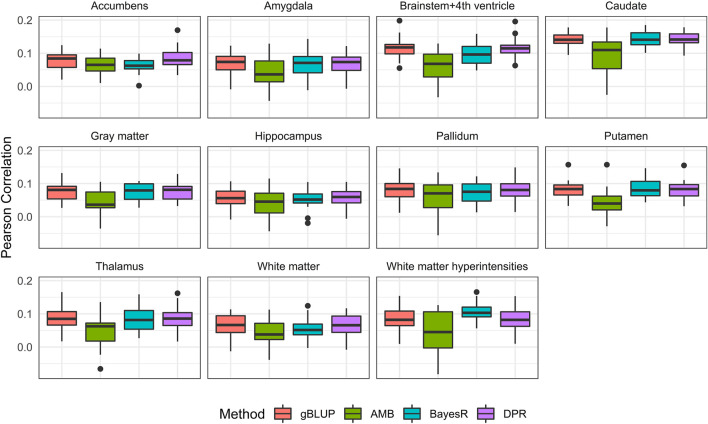
Pearson correlation for eleven brain imaging traits that are predicted using genetic variants from the UKB data. Methods include genomic best linear unbiased prediction (gBLUP), adaptive MultiBLUP (AMB), the latent Dirichlet process regression (DPR) and BayesR.

Without covariates considered, the Pearson correlation and MSE based on the 20-fold cross validation were shown in Figure 4 and Supplementary Figure S1, respectively. The prediction accuracy for external validation (i.e., ADNI data) was shown in Table 3 (Pearson correlation) and Supplementary Table S5 (MSE). Not surprisingly, all prediction models failed to capture most of the estimated heritability. Consistent with the trends seen from the Manhattan plots (Figure 3), the most computationally efficient gBLUP model performed the best or similar to the best model for all of the brain imaging traits based on both internal cross-validation and external data validation. We had noticed that both the cross-validation and external validation results were similar for most of the traits, except for the Gray matter and Brainstem+4^th^ ventricle. In addition, we found that the performance of AMB can vary substantially for each cross-validation, indicating AMB was much less robust than the other methods. As shown in Figure 3, there were no regions that were significantly different from the others for all these brain imaging traits. Therefore, the identification of significant regions adopted by AMB can introduce additional variability, leading to a relatively unstable prediction model. The computational resources needed for each prediction model were shown in Supplementary Table S6. Since there was no apparent gain in prediction accuracy for more complicated models, the simple and computationally efficient gBLUP model was more appropriate for the analyses of these brain imaging traits.

**TABLE 3:** The Pearson correlation for cross-validation and external validation for eleven brain imaging traits that are predicted using genetic variants only.

Phenotype	gBLUP		AMB		BayesR		DPR	
	UKB^a^	ADNI^b^	UKB^a^	ADNI^b^	UKB^a^	ADNI^b^	UKB^a^	ADNI^b^
*Hippocampus*	0.0561	0.0705	0.0456	0.0710	0.0519	0.0731	0.0596	0.0719
Accumbens	0.0843	0.0708	0.0652	0.0025	0.0627	0.0540	0.0790	0.0683
Amygdala	0.0740	0.0462	0.0364	0.0480	0.0711	0.0091	0.0736	−0.0199
Caudate	0.1407	0.1597	0.1098	−0.0182	0.1408	0.1844	0.1414	0.1561
Pallidum	0.0840	0.0959	0.0706	0.0473	0.0755	0.0933	0.0807	0.0897
Putamen	0.0836	0.0678	0.0400	0.0730	0.0796	0.1274	0.0835	0.0623
Thalamus	0.0849	0.0890	0.0625	−0.0621	0.0816	0.0612	0.0857	0.0888
Gray matter	0.0808	−0.0380	0.0363	−0.0703	0.0794	−0.0157	0.0812	0.0002
White matter	0.0664	0.1329	0.0380	−0.0987	0.0514	0.0933	0.0658	0.0407
White matter hyperintensity	0.0820	0.0766	0.0452	0.1238	0.1031	0.0800	0.0819	−0.0661
Brainstem+4th ventricle	0.1178	0.0499	0.0682	0.0847	0.0964	0.0408	0.1148	0.0528

a The average Pearson correlation calculated based on the 20-fold cross-validation using UKB data.

b The Pearson correlation calculated based on ADNI, data using the models built with UKB data.

**FIGURE 5:**
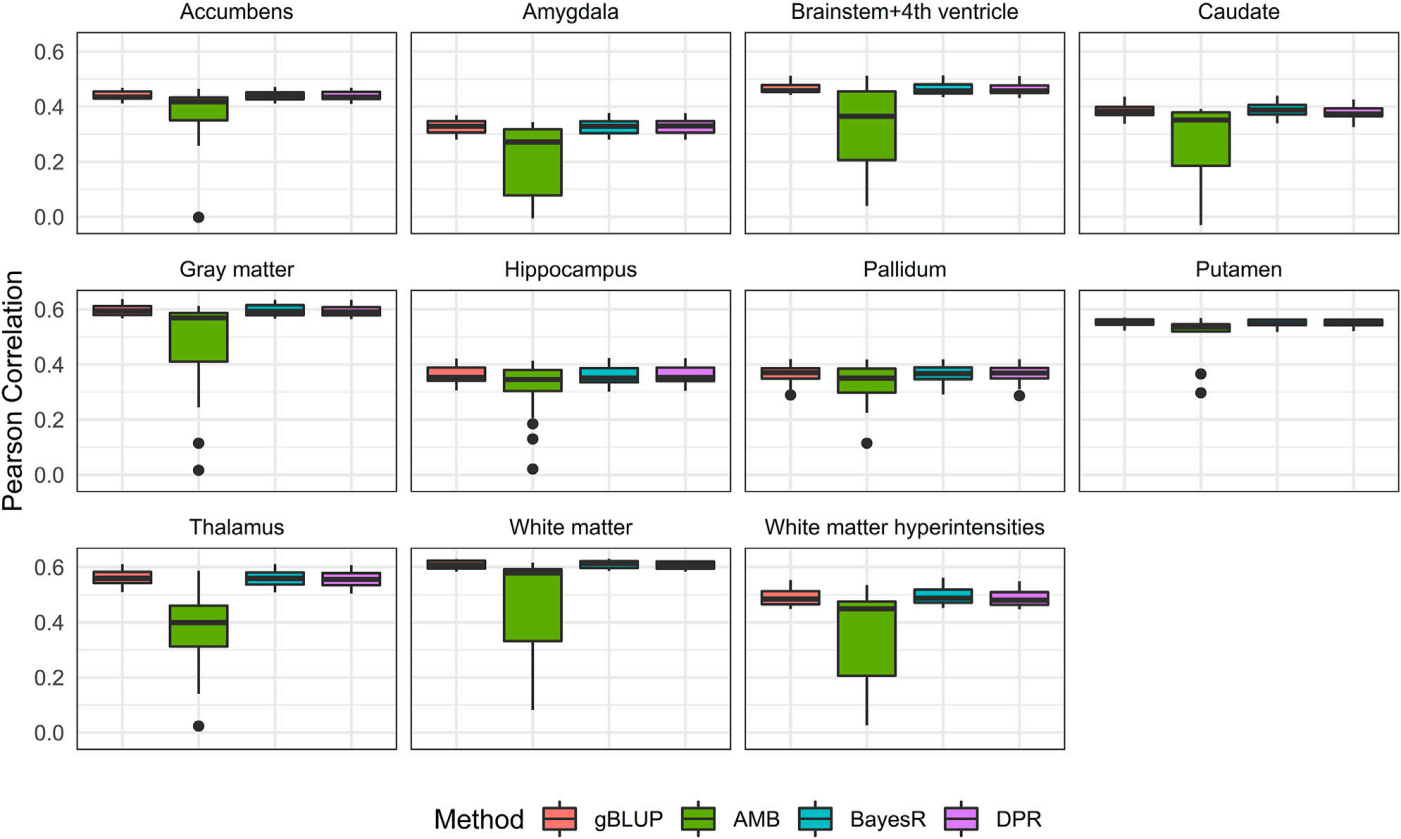
Pearson correlation for eleven brain imaging traits that are predicted using genetic variants and demographic variables (age, sex and education) in the UKB data. Methods include genomic best linear unbiased prediction (gBLUP), adaptive MultiBLUP (AMB), the latent Dirichlet process regression (DPR) and BayesR.

With covariates (i.e., age, sex and education) considered, the prediction accuracy based on both 20-fold cross validation and external validation were shown in Figure 5 and Table 4. As expected, the known AD risk factors substantially improved the risk prediction models. For Pearson correlation, similar to those observed from models without covariates, gBLUP, BayesR and DPR performed similarly, whereas AMB had much larger variability. For MSE (Supplementary Figure S2 and Supplementary Table S7), we had noticed that DPR had larger MSE than the other methods, and this may be due to the two-step procedures, where the parameters in DPR were not jointly inferred. We also noticed that the prediction models had different ability in predicting these AD-related traits. For example, for both grey and white matter, the Pearson correlation between predicted and observed values from cross-validation was all around 0.6, whereas it was less than 0.4 for amygdala and hippocampus. Regarding the external validation (Table 4 and Supplementary Table S7), the prediction accuracy generally tended to be similar or worse than that from the cross-validation. Nevertheless, for all these brain imaging traits, the simplest gBLUP models achieved fairly good prediction performance with minimum requirement of computational resources, which were shown in Supplementary Table S8.

**TABLE 4:** The Pearson correlation for cross-validation and external validation for eleven brain imaging traits that are predicted using genetic variants and demographic variables (age, sex and education).

Phenotype	gBLUP		AMB		BayesR		DPR	
	UKB^a^	ADNI^b^	UKB^a^	ADNI^b^	UKB^a^	ADNI^b^	UKB^a^	ADNI^b^
*Hippocampus*	0.3534	0.3654	0.3455	0.3674	0.3505	0.3661	0.3533	0.3654
Accumbens	0.4348	0.4108	0.4181	0.0773	0.4372	0.4103	0.4345	0.4127
Amygdala	0.3293	0.3189	0.2720	0.2386	0.3292	0.3144	0.3300	0.3195
Caudate	0.3836	0.2457	0.3519	0.1790	0.3876	0.2576	0.3735	0.2262
Pallidum	0.3706	0.4264	0.3504	0.2860	0.3671	0.4258	0.3692	0.4252
Putamen	0.5532	0.4337	0.5357	0.3499	0.5480	0.4464	0.5524	0.4310
Thalamus	0.5597	0.4593	0.3993	0.3247	0.5595	0.4644	0.5558	0.4593
Gray matter	0.5942	0.5161	0.5693	0.5011	0.5930	0.5191	0.5908	0.5080
White matter	0.6075	0.5568	0.5788	0.5582	0.6124	0.5583	0.6074	0.5511
White matter hyperintensity	0.4843	0.4418	0.4491	0.4444	0.4879	0.4478	0.4805	0.4417
Brainstem+4th ventricle	0.4578	0.5191	0.3650	0.1610	0.4571	0.5183	0.4570	0.5230

a The average Pearson correlation calculated based on the 20-fold cross-validation using UKB data.

b The Pearson correlation calculated based on ADNI, data using the models built with UKB data.

## Discussion

Over the past decades, the prediction of complex traits/diseases has gained tremendous popularities, and many analytical methods have been developed for such purposes (Yang et al., 2010; Visscher et al., 2013; Speed and Balding, 2014; Moser et al., 2015; Zeng and Zhou, 2017). While it is widely accepted that the underlying genetic architectures are trait-dependent and can substantially affect the performance of risk prediction models, it is unclear what information can be informative for choosing appropriate prediction models. Although prediction models that are flexible in modeling traits with various genetic architecture have the potential to achieve better performance, they generally are more computationally expensive and can have reduced prediction performance when it is over parameterized. Indeed, the simplest gBLUP model can achieve a similar level of performance as those complex models for many traits, but with a much-reduced request for computational resources. Brain imaging traits are important for AD detection, and their prediction based on both genetic and demographic risk factors can facilitate the ongoing treatment and intervention for AD. However, it is not clear how to best model them, which is not only because their underlying genetic causes are unknown, but also lack of the consensus on selecting the appropriate prediction models. Therefore, in this research, we first explored the relationship between genetic architecture and prediction accuracy of LMM-based models *via* visualizing the Manhattan plots using WTCCC data, and then constructed prediction models for eleven brain imaging traits based on both the ADNI and UKB data. Based on our exploration, we found that the simple Manhattan plots obtained from GWAS can be informative for prediction model selection, and the simple and computationally efficient gBLUP model can achieve the best or close to the best performance for most of the traits except those that showed highly significant and clustered spiked signals on the Manhattan plots. gBLUP achieves an appealing balance between computational tractability and prediction accuracy for the prediction of AD-related brain imaging traits that are likely to be highly polygenic.

How to choose an appropriate prediction model for diseases/traits? Existing prediction models normally differ in the assumptions on effect size distributions, and their performance is generally sensitively to the underlying genetic architecture that is usually unknown in advance. The most widely adopted assumptions are the sparse effect models and the infinitesimal effect models, where the former assumes that a few SNPs have large predictive effects and the majority of the SNPs are noise. The latter assumes that all SNPs have small to moderate effects for predictions. Correspondingly, the risk prediction models that adopt the sparsity assumption are more suitable for oligogenic diseases. This is mainly because under the oligogenic disease model, traits are expected to be predicted with a few SNPs with large predictive effects. They force the small effect size to be zero to improve the robustness of the model, and thus they naturally lose the ability to capture their effects. On contrary, prediction models that employ the infinitesimal effect assumption have natural advantage in capturing SNPs with small to moderate effect sizes, but they may lose power when the effect sizes for SNPs are much larger than expected. In practice, the underlying genetic mechanisms for most complex traits are unknown in advance, and thus it can be challenging to choose appropriate analytical methods. Although models that can accommodate a wide range of disease model assumptions are preferred in many applications, it is not guaranteed that flexible models are always more accurate than a simple one, let alone its heavy computation. Therefore, it is crucial to establish simple rules to facilitate model selection. In this research, we have found that the simple Manhattan plots obtained based on GWAS can be informative for inferring the appropriate model assumptions. For example, by exploring the Manhattan plots for T1D and RA that have spiked signals from some SNPs located nearby, it is highly unlikely that T1D and RA just have only the infinitesimal effects, and thus gBLUP model is not appropriate for these diseases. Similarly, the Manhattan plots for prediction of BD, CAD, HT and T2D barely show any genome-wide significant SNPs, thus the sparsity regression models are unlikely to work well, and the LMM-based models that allow SNPs having different effect size distributions are unlikely to benefit from introducing the additional flexibility. For CD that had a few isolated associated SNPs, the model that can capture both the infinitesimal effects and spiked isolated predictive effects is appropriate.

While Manhattan plots can facilitate the identification of traits with polygenic architecture that describes diseases were influenced by many SNPs with small effects, they are not capable of differentiating the oligogenic model and omnigenic model that assumes the gene regulatory networks are comprised of a small amount of core and highly significant disease-associated SNPs and a large amount of non-significant but predictive SNPs (Badano and Katsanis, 2002; Boyle et al., 2017). Therefore, we recommend for traits that do not exhibit any significant signals from the Manhattan plots, a simple gBLUP model is sufficient as allowing additional flexibility is not only unlikely to benefit the prediction accuracy, but also substantially increase the computational complexity (Supplementary Table S3 and Supplementary Table S6). However, for traits that have highly significant SNPs from the Manhattan plots, we recommend using DPR that is flexible in capturing both polygenic and oligogenic effects (i.e., the omnigenic effects). Under these circumstances, we do not recommend using the simple gBLUP model due to the presence of highly significant SNPs, nor do we recommend the sparsity regression models as they can have substantially worse performance for traits with omnigenic architecture. While the Manhattan plots can be influenced by both effect sizes of the SNPs and the sample size of the study, it can be informative and provide practical guidelines in choosing the appropriate prediction models for the given dataset. We noticed that as the sample size grows, more significant SNPs can be detected and the Manhattan plots can look differently from study to study. Therefore, we recommend to choose the appropriate prediction models based on the Manhattan plots of the training data at hand. In addition, different GWAS models could also have an impact on the appearance of the Manhattan plots (GWAS results under dominant and recessive models are shown in Supplementary Figure S3 and Supplementary Figure S4). Therefore, it is important to align the model assumption (e.g., additive, dominant or recessive) used in GWAS with the prediction models.

For the prediction analysis of AD-related brain imaging traits, the Manhattan plots from these eleven traits highly suggest the polygenic model. As expected, gBLUP model has the best or close to the best prediction performance for all these traits (Table 3 and Figure 4). With covariates (i.e., age, gender and education) incorporated, the prediction accuracy has increased substantially for all models, which is consistent with previous study (Ferreira et al., 2017). We have found that the prediction accuracy for these eleven diseases differs a lot, with Pearson correlation ranging from 0.27 to 0.61. We estimated the heritability for all these traits based on UKB data and found that there was a linear relationship between the estimated heritability and prediction accuracy (supplementary Table S9), which is in line with Yang *et al.* (Yang and Zhou, 2020).

Brain structures are believed to be moderate to high heritable (Carmelli D et al., 1998; Braskie et al., 2011; Satizabal et al., 2019). However, even with covariates incorporated, their prediction models do not have high accuracy. This is consistent with existing studies, which showed that common variants can only explain a small proportion of heritability for brain-related traits (Hibar et al., 2015). It is worth investigating the contributions of rare variants for prediction, as they can play important roles in brain-related traits (Korte and Farlow, 2013) (Manolio et al., 2009). While rare variants were measured by the ADNI study, they are not measured by UKB and future studies are needed to account for their effects. Gene-environmental interaction (G×E) exists in many common diseases and traits (Frost et al., 2016; Wang et al., 2017). SNPs×age (Bellou et al., 2020), SNPs×sex (Cacciottolo et al., 2016) and SNPs×education (Wang et al., 2017) have all been reported for AD. Therefore, it would be a future direction of our research to consider G×E interaction for the prediction of brain traits. Brain imaging traits tend to be correlated (Carmelli D et al., 1998; Glahn et al., 2007; Bogdan et al., 2017; Satizabal et al., 2019; Matoba et al., 2022), and thus joint modeling is another direction of our future research.

In summary, the performance of the LMM-based models is influenced by the underlying unknown genetic architecture. However, the simple Manhattan plots can be quite informative to facilitate model selection. For a given dataset, DPR that can capture both polygenic and oligogenic effects is recommended for traits with highly significant SNPs. For traits without obvious significant signals, a simple gBLUP model is sufficient, as it can get a good balance between accuracy and computation. We do not recommend the sparsity regression model even for traits that showed clustered spiked signals, and this is primarily due to the omnigenic architecture of many traits. For AD-related brain imaging traits that are likely to be polygenic as shown in Manhattan plots, we believe gBLUP is sufficient in modeling them and incorporating well-known demographic risk factors can further improve their prediction substantially.

## Data Availability

The data analyzed in this study is subject to the following licenses/restrictions: The datasets can be found at http://adni.loni.ucla.edu/ (ADNI), https://www.wtccc.org.uk/ (WTCCC) and https://www.ukbiobank.ac.uk/ (UK Biobank). They can be requested from ADNI, WTCCC and UK Biobank studies. Requests to access these datasets should be directed to ADNI: http://adni.loni.ucla.edu/; WTCCC: https://www.wtccc.org.uk/; and UK Biobank: https://www.ukbiobank.ac.uk/.
