# Supplementary material for: A guidance of model selection for genomic prediction based on linear mixed models for complex traits
Source: Front Genet. 2022 Oct 5;13:1017380. doi: 10.3389/fgene.2022.1017380 (PMC9581223; doi:10.3389/fgene.2022.1017380)
Supplement: Supplementary file 1 [file DataSheet1.PDF]

Supplementary Material

Supplementary Table S1. The number of individuals and SNPs for seven diseases in WTCCC.

Phenotype	Abbreviation	Individual number	SNPs number
Bipolar disease	BD	4862	67332
Coronary artery disease	CAD	4893	67339
Crohn's disease	CD	4888	67281
Hypertension	HT	4879	67959
Rheumatoid arthritis	RA	4882	68412
Type 1 diabetes	T1D	4926	67976
Type 2 diabetes	T2D	4873	67934

Supplementary Table S2. The number of individuals of eleven brain imaging traits in UKB and ADNI.

Phenotype	Individual	
	UKB	ADNI
Hippocampus	8348	657
Accumbens	8348	542
Amygdala	8348	542
Caudate	8348	542
Pallidum	8348	542
Putamen	8348	542
Thalamus	8348	542
Gray matter	8355	475
White matter	8355	475
White matter hyperintensity	7717	475
Brainstem+4th ventricle	8348	335

Supplementary Table S3. The computation resources for seven diseases in WTCCC.

Phenotype	gBLUP		AMB		BayesR		DPR	
	Time (hours)	Memory (GB)	Time (hours)	Memory (GB)	Time (hours)	Memory (GB)	Time (hours)	Memory (GB)
Bipolar disease	0.31	0.38	0.81	0.33	5.39	0.32	15.31	4.84
Coronary artery disease	0.31	0.38	0.88	0.33	5.80	0.32	14.88	4.87
Crohn's disease	0.30	0.38	0.81	0.33	5.38	0.32	21.28	4.86
Hypertension	0.32	0.38	1.09	0.33	5.76	0.32	15.48	4.91
Rheumatoid arthritis	0.31	0.38	0.86	0.33	5.69	0.33	21.93	4.93
Type 1 diabetes	0.36	0.40	0.95	0.33	5.43	0.33	17.89	4.95
Type 2 diabetes	0.32	0.38	0.86	0.33	5.73	0.32	20.91	4.89

Supplementary Table S4. The demographic information of eleven brain imaging traits in UKB and ADNI.

Phenotype	Age (Mean ± SD)		Sex (Male/Female)		Education (Lower/Middle/Higher)	
	UKB	ADNI	UKB	ADNI	UKB	ADNI
Hippocampus	55.33±7.44	73.30±6.85	4058/4289	370/286	539/3627/4181	3/221/432
Accumbens	55.33±7.44	72.97±6.78	4058/4289	297/244	539/3627/4181	2/183/356
Amygdala	55.33±7.44	72.97±6.78	4058/4289	297/244	539/3627/4181	2/183/356
Caudate	55.33±7.44	72.97±6.78	4058/4289	297/244	539/3627/4181	2/183/356
Pallidum	55.33±7.44	72.97±6.78	4058/4289	297/244	539/3627/4181	2/183/356
Putamen	55.33±7.44	72.97±6.78	4058/4289	297/244	539/3627/4181	2/183/356
Thalamus	55.33±7.44	72.97±6.78	4058/4289	297/244	539/3627/4181	2/183/356
White matter	55.34±7.44	72.65±7.22	4064/4290	255/219	539/3630/4185	0/158/316
White matter hyperintensity	55.34±7.44	72.65±7.22	4064/4290	255/219	539/3630/4185	0/158/316
White matter	55.34±7.41	72.65±7.22	3754/3962	255/219	506/3371/3839	0/158/316
Brainstem+4th ventricle	55.33±7.44	73.26±6.82	4058/4289	174/160	539/3627/4181	1/122/211

Supplementary Table S5. The mean square error (MSE) for cross-validation and external validation for eleven brain imaging traits that are predicted using genetic variants only.

Phenotype	gBLUP		AMB		BayesR		DPR	
	UKB[a]	ADNI[b]	UKB[a]	ADNI[b]	UKB[a]	ADNI[b]	UKB[a]	ADNI[b]
Hippocampus	0.9874	0.9936	1.0497	0.9974	0.9880	0.9934	0.9870	0.9934
Accumbens	1.0171	0.9948	1.0217	1.0387	1.0177	0.9954	0.9874	0.9966
Amygdala	1.0220	0.9963	1.0560	1.0220	1.0227	1.0008	1.0197	1.0047
Caudate	0.9336	0.9727	0.9518	1.3665	0.9345	0.9656	0.9384	0.9797
Pallidum	0.9810	0.9894	1.0173	1.0002	0.9809	0.9904	0.9805	0.9905
Putamen	0.9934	0.9947	1.0097	1.0046	0.9932	0.9831	0.9940	0.9944
Thalamus	1.0082	0.9903	1.0682	1.0572	1.0067	0.9946	1.0086	0.9982
Gray matter	1.0088	0.9757	1.0347	0.9759	1.0043	0.9821	1.0080	0.9778
White matter	0.9973	0.9824	1.0195	70.7709	0.9958	0.9896	0.9968	0.9971
White matter hyperintensity	1.0024	0.9951	1.0225	1.2426	0.9882	0.9967	1.0021	0.9945
Brainstem+4th ventricle	1.0031	0.9997	1.0761	232101.747	1.0035	0.9974	1.0027	0.9958

[a]: *The average mean square error calculated based on the 20-fold cross-validation using UKB data.*
[b]: *The mean square error calculated based on ADNI data using the models built with UKB data.*

Supplementary Table S6. The computation resources needed for cross-validation based on UKB for eleven brain imaging traits that are predicted using genetic variants only.

Phenotype	gBLUP		AMB		BayesR		DPR	
	Time (hours)	Memory (GB)	Time (hours)	Memory (GB)	Time (hours)	Memory (GB)	Time (hours)	Memory (GB)
Hippocampus	1.85	0.88	5.43	0.88	17.88	1.55	132.04	24.01
Accumbens	1.97	0.88	5.93	0.75	17.85	1.55	103.77	24.01
Amygdala	1.61	0.88	5.88	0.75	17.43	1.55	153.31	24.00
Caudate	1.93	0.88	5.78	0.88	18.90	1.55	131.42	24.01
Pallidum	1.94	0.88	5.75	0.88	18.04	1.55	131.84	24.01
Putamen	1.94	0.88	5.75	0.88	18.42	1.55	128.33	24.00
Thalamus	1.98	0.88	5.95	0.75	19.23	1.55	111.71	24.01
Gray matter	1.99	0.88	5.69	0.75	17.47	1.55	86.38	24.02
White matter	1.94	0.88	6.05	0.75	22.45	1.55	90.23	24.02
White matter hyperintensity	1.33	0.78	4.79	0.67	16.89	1.44	76.17	22.16
Brainstem+4th ventricle	1.94	0.88	5.90	0.75	17.69	1.55	124.24	24.01

Supplementary Table S7. The mean square error (MSE) for cross-validation and external validation for eleven brain imaging traits that are predicted using genetic variants and demographic variables (age, sex and education).

Phenotype	gBLUP		AMB		BayesR		DPR	
	UKB[a]	ADNI[b]	UKB[a]	ADNI[b]	UKB[a]	ADNI[b]	UKB[a]	ADNI[b]
Hippocampus	0.8451	1.1440	0.8877	1.1398	0.8456	1.1110	0.9662	0.9933
Accumbens	0.8079	1.6708	0.8659	3.3578	0.8087	1.7345	1.0011	0.9950
Amygdala	0.8902	0.8971	0.9555	0.9753	0.8910	0.9014	0.9994	0.9952
Caudate	0.8513	1.0146	0.9553	1.2344	0.8498	1.0049	0.9988	0.9856
Pallidum	0.8633	0.9141	0.8844	1.2187	0.8609	0.9287	1.0036	0.9914
Putamen	0.6787	1.2541	0.7084	1.3937	0.6790	1.2666	0.9794	0.9937
Thalamus	0.6874	1.3596	1.0106	1.6806	0.6890	1.3862	1.0023	0.9921
Gray matter	0.6320	1.4008	0.6784	1.3779	0.6333	1.4443	0.9793	0.9859
White matter	0.6328	0.7674	0.6838	0.7619	0.6317	0.7831	1.0055	0.9872
White matter hyperintensity	0.7575	2.0014	0.8477	1.9724	0.7596	2.0362	0.9975	0.9937
Brainstem+4th ventricle	0.7918	0.7380	0.9110	2.7556	0.7932	0.7417	0.9910	0.9934

[a]*: The average mean square error calculated based on the 20-fold cross-validation using UKB data.*
[b]*: The mean square error calculated based on ADNI data using the models built with UKB data.*

Supplementary Table S8. The computation resources needed for cross-validation based on UKB for eleven brain imaging traits that are predicted using genetic variants and demographic variables (age, sex and education).

Phenotype	gBLUP		AMB		BayesR		DPR	
	Time (hours)	Memory (GB)	Time (hours)	Memory (GB)	Time (hours)	Memory (GB)	Time (hours)	Memory (GB)
Hippocampus	5.50	0.75	6.16	0.75	23.41	1.59	1919.08	70.51
Accumbens	5.41	0.75	6.16	0.75	18.50	1.59	1955.62	70.51
Amygdala	5.38	0.75	6.16	0.75	17.61	1.59	1103.60	70.51
Caudate	5.50	0.75	6.21	0.75	17.66	1.59	1873.69	70.51
Pallidum	5.28	0.75	5.99	0.75	17.30	1.59	1538.55	70.51
Putamen	5.39	0.75	5.99	0.75	24.75	1.59	1849.06	70.51
Thalamus	5.38	0.75	5.98	0.75	17.68	1.59	1461.91	70.51
Gray matter	5.25	0.75	5.98	0.75	17.75	1.59	1784.57	70.56
White matter	5.36	0.75	6.05	0.75	17.35	1.59	1927.75	70.56
White matter hyperintensity	4.26	0.67	4.89	0.67	17.19	1.48	1195.48	65.23
Brainstem+4th ventricle	5.32	0.75	5.99	0.75	18.15	1.59	1421.85	70.51

Supplementary Table S9. The relationship between the estimated heritability and prediction accuracy of compared methods.

Methods	Binary traits [a]	Continuous traits [b]
gBLUP	0.88	0.87
AMB	0.70	0.76
BayesR	0.53	0.65
DPR	0.78	0.86

[a]*: the Pearson correlation between the estimated heritability and Area under the curve of compared methods in WTCCC among seven diseases.*

[b]*: the Pearson correlation between the estimated heritability and Pearson correlation of compared methods in UKB among eleven brain imaging traits.*

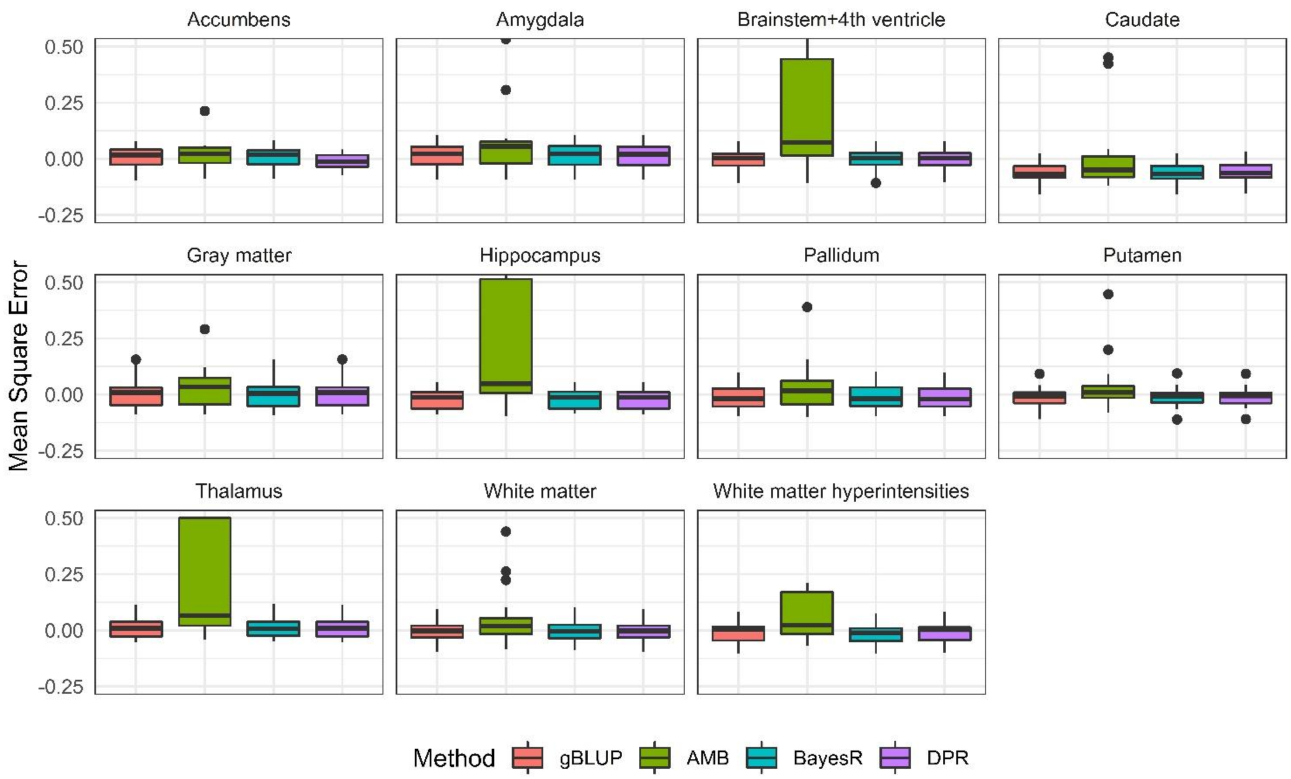

Supplementary Figure S1. The mean square error (MSE) for eleven brain imaging traits that are predicted using genetic variants from the UKB data. Natural logarithm was performed for MSE. All values greater than 0.5 are set to 0.5. Methods include genomic best linear unbiased prediction (gBLUP), adaptive MultiBLUP (AMB), the latent Dirichlet process regression (DPR) and BayesR.

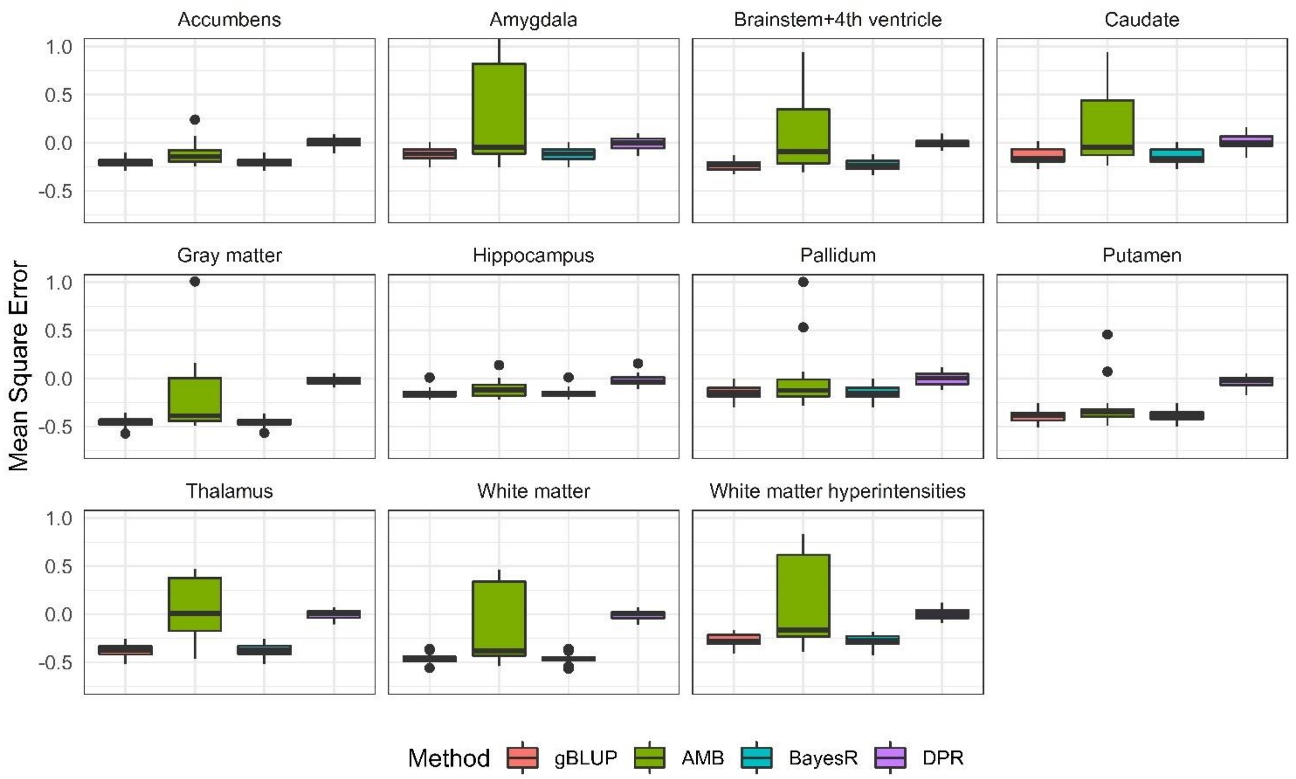

Supplementary Figure S2. The mean square error (MSE) for eleven brain imaging traits that are predicted using genetic variants and demographic variables (age, sex and education) in the UKB data. Natural logarithm was performed for all MSE. Methods include genomic best linear unbiased prediction (gBLUP), adaptive MultiBLUP (AMB), the latent Dirichlet process regression (DPR) and BayesR.

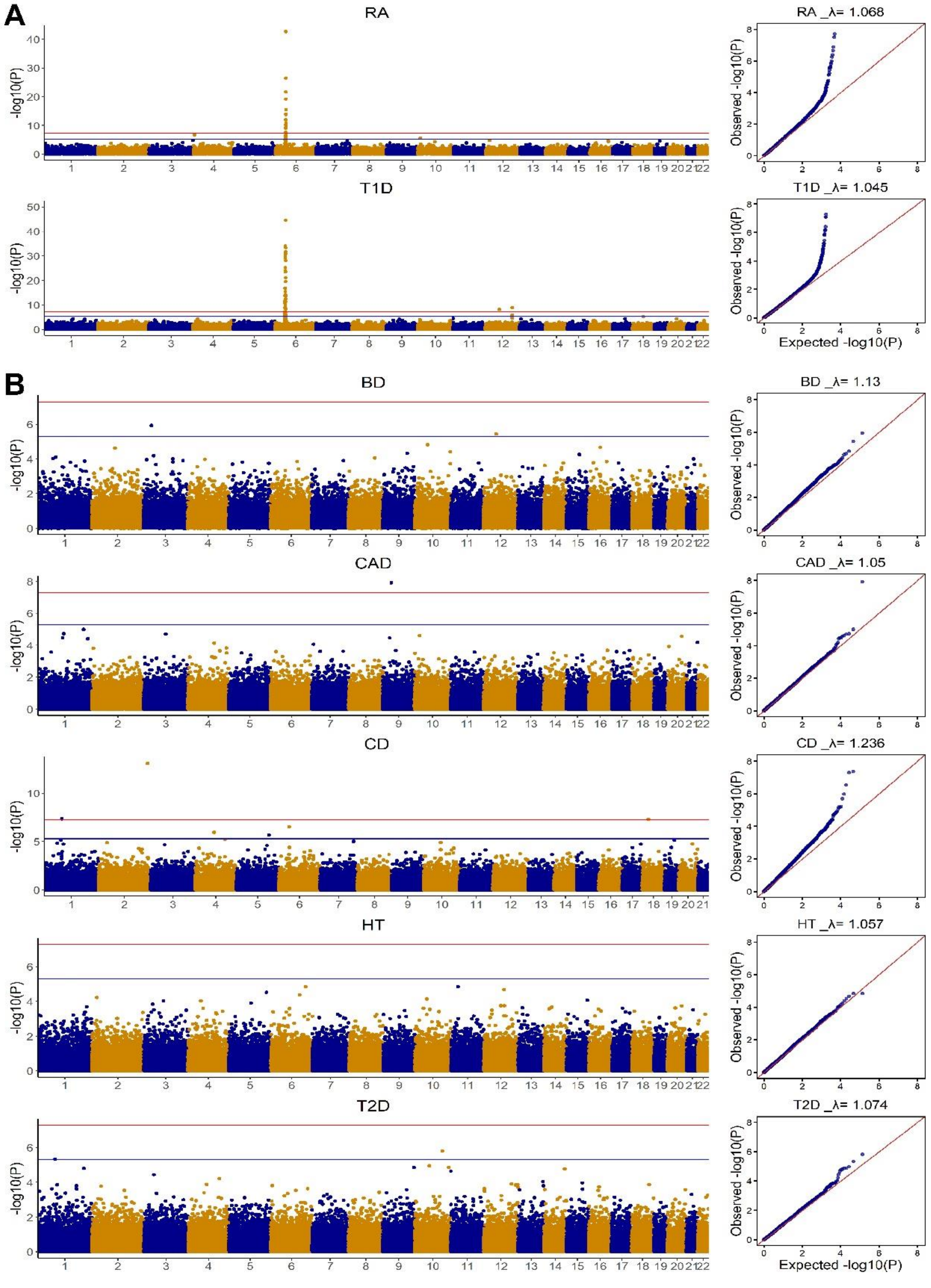

Supplementary Figure S3. Manhattan and Quantile-Quantile plots for seven diseases in WTCCC based on dominant model for Genome-wide association analysis. CD: Crohn’s disease; RA: Rheumatoid arthritis; T1D: Type 1 diabetes; BD: Bipolar disease; CAD: Coronary artery disease; HT: Hypertension; T2D: Type 2 diabetes. The λ closer to 1 indicates appropriate control of population structure.

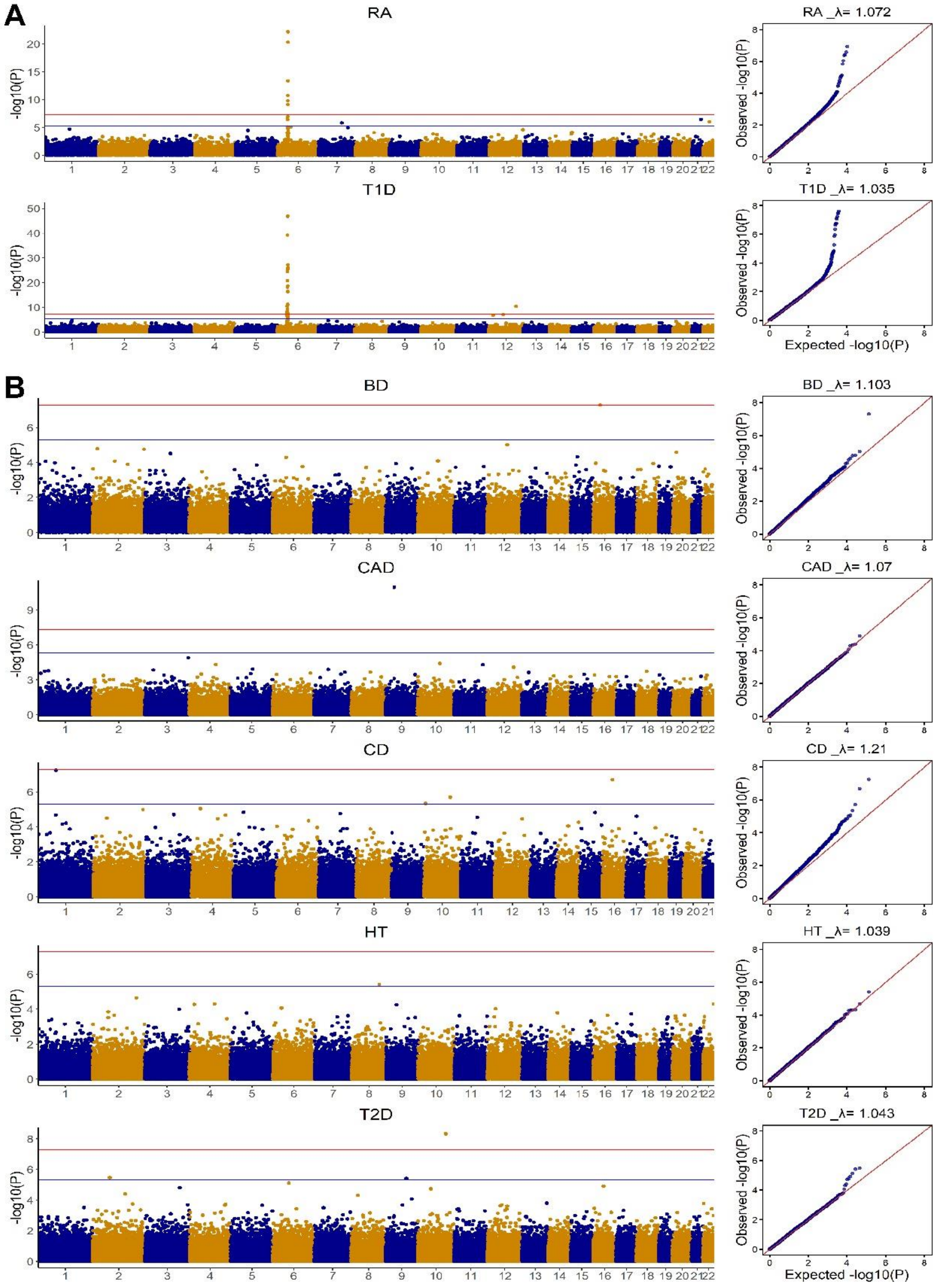

Supplementary Figure S4. Manhattan and Quantile-Quantile plots for seven diseases in WTCCC based on recessive model for Genome-wide association analysis. CD: Crohn’s disease; RA: Rheumatoid arthritis; T1D: Type 1 diabetes; BD: Bipolar disease; CAD: Coronary artery disease; HT: Hypertension; T2D: Type 2 diabetes. The λ closer to 1 indicates appropriate control of population structure.
